# Multiscale Stochastic Reaction–Diffusion Algorithms Combining Markov Chain Models with Stochastic Partial Differential Equations

**DOI:** 10.1007/s11538-019-00613-0

**Published:** 2019-06-04

**Authors:** Hye-Won Kang, Radek Erban

**Affiliations:** 1 Department of Mathematics and Statistics, University of Maryland Baltimore County, 1000 Hilltop Circle, Baltimore, MD 21250 USA; 2 Mathematical Institute, University of Oxford, Radcliffe Observatory Quarter, Woodstock Road, Oxford, OX2 6GG UK

**Keywords:** Stochastic reaction–diffusion systems, Chemical reaction networks, Markov chain, Gillespie algorithm, Multiscale modelling, Stochastic partial differential equations, 60J27, 60J28, 92C42, 35R60, 60J70, 65C05

## Abstract

Two multiscale algorithms for stochastic simulations of reaction–diffusion processes are analysed. They are applicable to systems which include regions with significantly different concentrations of molecules. In both methods, a domain of interest is divided into two subsets where continuous-time Markov chain models and stochastic partial differential equations (SPDEs) are used, respectively. In the first algorithm, Markov chain (compartment-based) models are coupled with reaction–diffusion SPDEs by considering a pseudo-compartment (also called an overlap or handshaking region) in the SPDE part of the computational domain right next to the interface. In the second algorithm, no overlap region is used. Further extensions of both schemes are presented, including the case of an adaptively chosen boundary between different modelling approaches.

## Introduction

Stochastic models of well-mixed chemical systems are traditionally formulated in terms of continuous-time Markov chains, which can be simulated using the Gillespie stochastic simulation algorithm (SSA) (Gillespie 1977) or its equivalent formulations (Gibson and Bruck 2000; Cao et al. 2004; Klingbeil et al. 2011). These algorithms provide statistically exact sample paths of stochastic chemical models described by the corresponding chemical master equation (CME). However, they can be computationally expensive for larger chemical systems, because they explicitly simulate each occurrence of each chemical reaction. A number of approaches have been developed in the literature to decrease the computational intensity of SSAs. Taking into account separation of timescales, chemical reaction networks can be simplified by model reduction before they are simulated (Kang 2012; Kang and Kurtz 2013; Kang et al. 2014; Kim et al. 2017; Kang et al. 2019). The idea of model reduction can also be used to develop computational methods which efficiently estimate quantities of interest from stochastic simulations (Cao et al. 2005a, b; Erban et al. 2006; Cotter et al. 2011). Another approach is to describe the molecular populations in terms of their concentrations that change continuously (rather than treating them as discrete random variables). This can be achieved by the chemical Langevin equation, which is a stochastic differential equation (SDE) acting as a bridge between discrete SSAs and deterministic reaction rate equations (Kurtz 1976, 1978; Gillespie 2000). Efficient algorithms which make use of the SDE approximations have been developed for the simulation of chemical systems especially when they include processes occurring on different timescales (Haseltine and Rawlings 2002; Salis and Kaznessis 2005; Griffith et al. 2006; Cotter and Erban 2016). More recently, the SDE approximations have been extensively used to develop hybrid algorithms which use boths SSAs and SDEs for different components of the studied systems (Liu et al. 2012; Ganguly et al. 2015; Duncan et al. 2016; Altintan et al. 2016). The chemical Fokker–Planck equation corresponding to the chemical Langevin equation can also be used to efficiently estimate quantities of interest from stochastic models (Erban et al. 2009; Cotter et al. 2013; Liao et al. 2015; Cucuringu and Erban 2017).

In this paper, we consider spatially distributed (reaction–diffusion) models which can be described in terms of the reaction–diffusion master equation (RDME) (Erban et al. 2007). A spatial domain is discretized into compartments (which are assumed to be well mixed), and diffusion is modelled as a jump process between neighbouring compartments (Erban and Chapman 2009; Kang et al. 2012b; Hu et al. 2014). In the literature, the RDME approach has been adapted to model and simulate spatially distributed systems using uniform meshes (equivalently, subvolumes or compartments) (Stundzia and Lumsden 1996; Elf et al. 2003; Lampoudi et al. 2009), nonuniform meshes (Bernstein 2005) or complex geometries (Isaacson and Peskin 2006). The resulting compartment-based model can be simulated by the Gillespie SSA. Compartment-based reaction–diffusion approaches have been used to model several intracellular processes, including Min oscillations in *E. coli* (Fange and Elf 2006; Arjunan and Tomita 2010), ribosome biogenesis (Earnest et al. 2015, 2016), actin dynamics in filopodia (Zhuravlev and Papoian 2009; Erban et al. 2014) and pattern formation in morphogen signalling pathways (Kang et al. 2012a). They have also been implemented in a number of software packages including MesoRD (Hattne et al. 2005), URDME (Engblom et al. 2009), STEPS (Wils and De Schutter 2009), SmartCell (Ander et al. 2004), Lattice Microbes (Roberts et al. 2013) and Smoldyn (Robinson et al. 2015). As in the case of the simulation of well-mixed systems, the Langevin approach provides an approximation of the compartment-based model which can reduce the computational intensity of simulations. Spatial Langevin approaches (Kalantzis 2009; Ghosh et al. 2015; Bhattacharjee et al. 2015) and stochastic partial differential equations (SPDEs) (Dogan and Allen 2011; Alexander et al. 2002, 2005; Atzberger 2010; Kim et al. 2017) have been suggested to model stochastic reaction–diffusion systems. A hybrid method has also been introduced using the Langevin approximation for diffusion coupled with the compartment-based model for reactions (Lo et al. 2016).

In the thermodynamic limit (of large populations), compartment-based models lead to reaction–diffusion partial differential equations (PDEs) which are written in terms of spatio-temporal concentrations of chemical species. This property can be exploited to design multiscale (hybrid) algorithms which use the compartment-based Markov chain model in a subset of the simulated system and apply reaction–diffusion PDEs in other parts (Kalantzis 2009; Ferm et al. 2010; Yates and Flegg 2015; Spill et al. 2015; Harrison and Yates 2016). Other hybrid methods have also been developed in the literature including methods which couple more detailed Brownian dynamics (molecular-based) approaches with the compartment-based method (Flegg et al. 2012; Klann et al. 2012; Flegg et al. 2015; Dobramysl et al. 2016) or with reaction–diffusion PDEs (Franz et al. 2013; Schaff et al. 2016; Bakarji and Tartakovsky 2017).

In this paper, we analyse two multiscale algorithms which couple compartment-based models with suitably discretized SPDEs. They can be used when a large number of molecules of some species are located in parts of the computational domain. In the region with a small number of molecules, we use a compartment-based model written as a continuous-time Markov chain. In other regions, we use SPDEs derived from the Markov process. The goal of this multiscale methodology is to get an approximation of the spatio-temporal statistics which we would obtain by running the underlying Markov chain model in the entire computational domain. The paper is organized as follows. In Sect. 2, we present the derivation of the SPDE description from the compartment-based model. In Sect. 3, two multiscale schemes are presented. An illustrative example with a static boundary between the SPDE and Markov chain subdomains is studied in Sect. 4. The algorithm is extended to a time-dependent interface in Sect. 5. In Sect. 6, we discuss an example with multiple species.

## From Continuous-Time Markov Jump Processes to Stochastic Partial Differential Equations

**Fig. 1:**
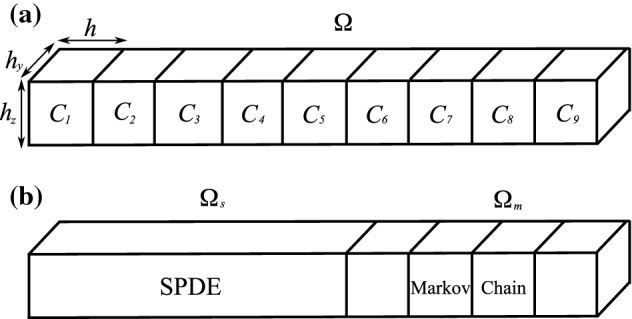
**a** A schematic illustration of the elongated domain $\varOmega$*for*$K=9$. **b** A schematic illustration of the multiscale setup

We consider a system of *N* chemically reacting species $$S_1,$$$$S_2$$, ..., $$S_N$$, which are diffusing (with diffusion constants $$D_i$$, $$i=1,2,\ldots ,N$$) in the bounded domain $$\varOmega \subset {{\mathbb {R}}}^3$$. We use a compartment-based stochastic reaction–diffusion model (Erban et al. 2007), i.e. we divide the domain $$\varOmega $$ into *K* compartments $$C_k$$, $$k=1,2,\ldots ,K$$, and model the diffusion as a jump process between neighbouring compartments. In order to simplify the analysis, we consider that $$\varOmega $$ is an elongated pseudo-one-dimensional domain $$\varOmega = [0,Kh] \times [0,h_y] \times [0,h_z]$$, where $$h,h_y,h_z>0$$, as shown in Fig. 1a. Compartments are rectangular cuboids with the volume $$hh_yh_z$$ where $$C_k = [(k-1)h,kh] \times [0,h_y] \times [0,h_z]$$ for $$k=1,2,\ldots ,K$$. Let $$Z_i^k(t)$$, $$i=1,2\ldots ,N$$, $$k=1,2,\ldots ,K$$, be the number of molecules of the *i*th chemical species in the *k*th compartment at time *t*. Then, $${{\mathbf {Z}}}^k(t)$$ is an *N*-dimensional column vector with each component representing the number of molecules of the corresponding species in the *k*th compartment at time *t*. We define

$$\begin{aligned} {{\mathbf {Z}}}(t) = \left( {{\mathbf {Z}}}^1(t)^{\mathrm {T}}, {{\mathbf {Z}}}^2(t)^{\mathrm {T}}, \ldots , {{\mathbf {Z}}}^K(t)^{\mathrm {T}} \right) ^{\mathrm {T}}, \end{aligned}$$

which is a *KN*-dimensional column vector and $\mathrm {T}$ denotes the transpose of a vector. We assume that the chemical system is subject to *M* chemical reactions with ${\varvec{\zeta }}_j$, $j=1,2,\ldots ,M$, being the corresponding *N*-dimensional stoichiometric vector. Let ${\varvec{\zeta }}_j^k$, $j=1,2\ldots ,M$, $k=1,2,\ldots ,K$, be a *KN*-dimensional stoichiometric vector which gives a net molecule change during each occurrence of the *j*th reaction in the *k*th compartment. Let ${\varvec{\nu }}_{-,i}^k$ (resp. ${\varvec{\nu }}_{+,i}^k$), $i=1,2,\ldots ,N$, $k=1,2,\ldots , K$ be a *KN*-dimensional stoichiometric vector which gives a net molecule change during diffusion of the *i*th species from the *k*th compartment to the $(k-1)$th (resp. $(k+1)$th) compartment. Let

$$\begin{aligned} \lambda _j^k: [0,\infty )^N \rightarrow [0,\infty ), \qquad j=1,2\ldots ,M, \quad k=1,2,\ldots ,K, \end{aligned}$$

be the propensity function of the *j*th chemical reaction in the *k*th compartment, i.e. $\lambda _j^k({{\mathbf {Z}}}^k(t)) \, \mathrm {d}t$ is the probability that the *j*th reaction occurs in the *k*th compartment during the time $[t,t+\mathrm {d}t)$ given that the current state at time *t* is ${{\mathbf {Z}}}^k(t)$. We denote by $R_j^k(t)$, $j=1,2\ldots ,M$, $k=1,2,\ldots ,K$, a random process which counts the number of times the *j*th reaction occurs in the *k*th compartment up to time *t*. Then,

1 $$\begin{aligned} R_j^k(t) = Y_j^k\left( \int _0^t \lambda _j^k({{\mathbf {Z}}}^k(s)) \,\text{ d }s\right) , \end{aligned}$$

where $Y_j^k$ are independent unit Poisson processes. We define $R_{-,i}^{k}(t)$ (resp. $R_{+,i}^{k}(t)$), $i=1,2,\ldots ,N$, $k=2,3,...,K$ (resp. $k=1,2,...,K-1$), random processes counting the numbers of times that one molecule of the *i*th species in the *k*th compartment diffuses to the $(k-1)$th compartment (resp. to the $(k+1)$th compartment) up to time *t*. Then,

2 $$\begin{aligned} R_{\pm ,i}^k(t) = Y_{\pm ,i}^k \left( \int _0^t\frac{D_i}{h^2} \, Z_i^k(s) \,\text{ d }s\right) , \end{aligned}$$

where $Y_{\pm ,i}^k$ are independent unit Poisson processes. The governing equation for the state vector ${{\mathbf {Z}}}(t)$ is

3 $$\begin{aligned} \begin{aligned} {{\mathbf {Z}}}(t)&= {{\mathbf {Z}}}(0) + \sum _{k=1}^K \sum _{j=1}^{M} R_j^k(t) \,{\varvec{\zeta }}_j^k + \sum _{k=2}^{K} \sum _{i=1}^N R_{-,i}^k(t) \,{\varvec{\nu }}_{-,i}^k + \sum _{k=1}^{K-1} \sum _{i=1}^N R_{+,i}^k(t) \,{\varvec{\nu }}_{+,i}^k. \end{aligned} \end{aligned}$$

When the propensities are large (Kurtz 1978), the counting processes in Eqs. (1)–(2) can be approximated as

$$\begin{aligned} R_j^k(t)\approx & {} \int _0^t\lambda _j^k({{\mathbf {Z}}}^k(s))\,\text{ d }s+ \int _0^t \sqrt{\lambda _j^k({{\mathbf {Z}}}^k(s))}\,\text{ d }W_j^k(s),\\ R_{\pm ,i}^k(t)\approx & {} \int _0^t\frac{D_i}{h^2} \, Z_i^k(s)\,\text{ d }s+ \int _0^t \sqrt{\frac{D_i}{h^2} \, Z_i^k(s)}\,\text{ d }W_{\pm ,i}^k(s), \end{aligned}$$

where $W_j^k$ and $W_{\pm ,i}^k$ are standard Brownian motions. Using ${\varvec{\nu }}_{+,i}^{k}=-{\varvec{\nu }}_{-,i}^{k+1}$ for $k=1,2,\ldots ,K-1$ and changing the index $(k+1) \rightarrow k$ in the last term of Eq. (3), governing Eq. (3) can be approximated by the following SDE (Gillespie 2000; Kurtz 1978)

4 $$\begin{aligned} {{\mathbf {Z}}}(t)= & {} {{\mathbf {Z}}}(0) + \sum _{k=1}^K\sum _{j=1}^M \left( \int _0^t \lambda _j^k({{\mathbf {Z}}}^k(s)) \,\text{ d }s+ \int _0^t \sqrt{\lambda _j^k({{\mathbf {Z}}}^k(s))} \,\text{ d }W_j^k(s) \right) {\varvec{\zeta }}_j^k \nonumber \\&+ \sum _{k=2}^K\sum _{i=1}^N \left\{ \int _0^t \frac{D_i}{h^2} \left( Z_i^k(s)-Z_i^{k-1}(s)\right) \,\text{ d }s+ \int _0^t \sqrt{\frac{D_i}{h^2} \,Z_i^k(s)} \,\text{ d }W_{-,i}^k(s) \right. \nonumber \\&\left. - \int _0^t \sqrt{\frac{D_i}{h^2} \,Z_i^{k-1}(s)} \,\text{ d }W_{+,i}^{k-1}(s) \right\} {\varvec{\nu }}_{-,i}^k. \end{aligned}$$

Since $W_{-,i}^{k}$ and $W_{+,i}^{k-1}$ terms always appear together in Eq. (4), and since the sum of independent normal random variables is normally distributed, Eq. (4) can be rewritten as

5 $$\begin{aligned} \begin{aligned} {{\mathbf {Z}}}(t)&= {{\mathbf {Z}}}(0) + \sum _{k=1}^K\sum _{j=1}^M \left( \int _0^t \lambda _j^k({{\mathbf {Z}}}^k(s)) \,\text{ d }s+ \int _0^t \sqrt{\lambda _j^k({{\mathbf {Z}}}^k(s))} \,\text{ d }W_j^k(s) \right) {\varvec{\zeta }}_j^k\\&\quad +\, \sum _{k=2}^K\sum _{i=1}^N \left\{ \int _0^t \frac{D_i}{h^2} \left( Z_i^k(s)-Z_i^{k-1}(s)\right) \,\text{ d }s\right. \\&\quad \left. +\, \int _0^t \sqrt{\frac{D_i}{h^2}\left( Z_i^{k}(s)+Z_i^{k-1}(s)\right) } \, \text{ d }W_i^{k-1}(s) \right\} {\varvec{\nu }}_{-,i}^k, \end{aligned} \end{aligned}$$

where $W_i^{k-1}$ is a standard Brownian motion. Let $V_h = hh_yh_z$ be the volume of each compartment, and define ${{\mathbf {c}}}(t) = {{\mathbf {Z}}}(t)/V_h$ as a concentration vector for species at time *t*. Define

$$\begin{aligned} {\widehat{\lambda }}_j^{k,h}({{\mathbf {c}}}^k(t)) = \frac{\lambda _j^k( {{\mathbf {Z}}}^k(t) )}{V_h}, \qquad \text{ where } \quad {{\mathbf {c}}}^k(t) = \frac{{{\mathbf {Z}}}^k(t)}{V_h}. \end{aligned}$$

Dividing Eq. (5) by $V_h$, we get

6 $$\begin{aligned} \begin{aligned} {{\mathbf {c}}}(t)&= {{\mathbf {c}}}(0) + \sum _{k=1}^K \sum _{j=1}^{M} \left( \int _0^t {\widehat{\lambda }}_j^{k,h}({{\mathbf {c}}}^k(s)) \,\text{ d }s+ \int _0^t \frac{1}{\sqrt{V_h}}\sqrt{{\widehat{\lambda }}_j^{k,h}({{\mathbf {c}}}^k(s))} \,\text{ d }W_j^k(s) \right) {\varvec{\zeta }}_j^k\\&\quad +\, \sum _{k=2}^K\sum _{i=1}^N \left\{ \int _0^t \frac{D_i}{h^2} \left( c_i^{k}(s)-c_i^{k-1}(s) \right) \text{ d }s\right. \\&\quad \left. +\, \int _0^t \frac{1}{\sqrt{V_h}} \sqrt{\frac{D_i}{h^2}\left( c_i^{k}(s)+c_i^{k-1}(s)\right) } \,\text{ d }W_i^{k-1}(s) \right\} {\varvec{\nu }}_{-,i}^k, \end{aligned} \end{aligned}$$

where $c_i^k(t) = Z_i^k(t)/V_h$. The second part of Eq. (6) is consistent with the discretized Langevin scheme for a diffusion equation, as studied in Alexander et al. (2002). We rewrite Eq. (6) using the fact that reaction happens among species in the same compartment and that diffusion occurs between neighbouring compartments. Differentiating Eq. (6), the concentration of the chemical species in the *k*th compartment satisfies

7 $$\begin{aligned} \begin{aligned} \text{ d }{{\mathbf {c}}}^k(t)&= \sum _{j=1}^{M} \left( {\widehat{\lambda }}_j^{k,h}({{\mathbf {c}}}^k(t)) \,\text{ d }t+ \frac{1}{\sqrt{V_h}}\sqrt{{\widehat{\lambda }}_j^{k,h}({{\mathbf {c}}}^k(t))} \,\text{ d }W_j^k(t) \right) {\varvec{\zeta }}_j\\&\quad +\, \frac{{{\mathbf {D}}}}{h^2} \left\{ \left( {{\mathbf {c}}}^{k+1}(t)-{{\mathbf {c}}}^{k}(t) \right) \chi _{\{k\ne K\}} -\left( {{\mathbf {c}}}^{k}(t)-{{\mathbf {c}}}^{k-1}(t) \right) \chi _{\{k\ne 1\}} \right\} \text{ d }t\\&\quad +\, \frac{1}{\sqrt{V_h}} \frac{1}{h} \left\{ \text{ d }{{\mathbf {W}}}^{k}(t) \sqrt{{{\mathbf {D}}}\left( {{\mathbf {c}}}^{k+1}(t)+{{\mathbf {c}}}^{k}(t)\right) } \, \chi _{\{k\ne K\}} \right. \\&\quad \left. -\,\text{ d }{{\mathbf {W}}}^{k-1}(t) \sqrt{{{\mathbf {D}}}\left( {{\mathbf {c}}}^{k}(t)+{{\mathbf {c}}}^{k-1}(t)\right) } \, \chi _{\{k\ne 1\}} \right\} , \end{aligned} \end{aligned}$$

where ${{\mathbf {W}}}^k(t)$ are $N\times N$ diagonal matrices with $W_i^k(t)$ on its diagonal for $i=1,2,\ldots ,N$, $k=1,2,\ldots ,K-1$ and $\chi _{\{\cdot \}}$ is an indicator function. In Eq. (7), ${\varvec{\zeta }}_j$ is an *N*-dimensional stoichiometric vector of the *j*th reaction for $j=1,2,\ldots ,M$, and ${{\mathbf {D}}}$ is a $N \times N$ diagonal matrix which has diffusion constants of individual species on its diagonal, i.e.

$$\begin{aligned} {{\mathbf {D}}}= \left[ \begin{array}{ccccc} D_1 &{} 0 &{} \ldots &{} 0\\ 0 &{} D_2 &{} \dots &{} 0\\ \vdots &{} \vdots &{} \ddots &{} 0\\ 0 &{} 0 &{} \dots &{} D_N\\ \end{array}\right] . \end{aligned}$$

We approximate white noise processes in Eq. (7) as

$$\begin{aligned} \frac{1}{\sqrt{h}}\frac{\text{ d }W_j^k(t)}{\text{ d }t} \approx \eta _j(x,t), \qquad \frac{1}{\sqrt{h}}\frac{\text{ d }{{\mathbf {W}}}^k(t)}{\text{ d }t} \approx {\varvec{\xi }}(x,t), \end{aligned}$$

where $\eta _j(x,t)$, $j=1,2,\ldots ,M,$ are spatio-temporal white noise processes (Walsh 1986), i.e.

$$\begin{aligned} \int _{x^\prime }^{x^\prime + \varDelta x} \int _{t^\prime }^{t^\prime + \varDelta t} \eta _j(x,t) \, \text{ d }x\, \text{ d }t,\quad x^\prime \in [0,Kh], \;\, t^\prime \in [0,\infty ), \end{aligned}$$

is normally distributed with zero mean and variance $\varDelta x \varDelta t$. Matrices ${\varvec{\xi }}(x,t)$ are diagonal $N \times N$ matrices where diagonal entries are independent spatio-temporal white noise processes. Then, Eq. (7) is a solution of a discretized version of a SPDE in space which can be formally written in the following form

8 $$\begin{aligned} \frac{\partial {{\mathbf {c}}}(x,t)}{\partial t}= & {} \sum _{j=1}^{M} {\widehat{\lambda }}_j({{\mathbf {c}}}(x,t),x) \,{\varvec{\zeta }}_j + {{{\mathbf {D}}}}\frac{\partial ^2 {{\mathbf {c}}}(x,t)}{\partial x^2} + \sum _{j=1}^{M} \sqrt{\frac{{\widehat{\lambda }}_j({{\mathbf {c}}}(x,t),x)}{h_yh_z}} \,{\varvec{\zeta }}_j \, \eta _j(x,t) \nonumber \\&{+} \frac{\partial }{\partial x}\left[ {{\varvec{\xi }}(x,t)} \, \sqrt{\frac{2 {{{\mathbf {D}}}} \, {{\mathbf {c}}}(x,t)}{h_yh_z}} \right] , \end{aligned}$$

where ${{\mathbf {c}}}(x,t)$ is a spatio-temporal concentration related to ${{\mathbf {c}}}^k(t)$ by

$$\begin{aligned} \frac{1}{h}\int _{(k-1)h}^{kh} {{\mathbf {c}}}(x',t)\, \mathrm {d}x' \approx {{\mathbf {c}}}^k(t). \end{aligned}$$

The reaction term ${\hat{\lambda }}{_j}: [0,\infty )^N\times [0,Kh]\rightarrow [0,\infty )$ in Eq. (8) is related to ${\hat{\lambda }}_j^{k,h}$ by

$$\begin{aligned} \frac{1}{h}\int _{(k-1)h}^{kh} {\hat{\lambda }}_j({{{\mathbf {c}}}(x',t)},x')\, \mathrm {d}x' {\,\approx \,} {\hat{\lambda }}_j^{k,h}({{{\mathbf {c}}}^k(t)}). \end{aligned}$$

Note that Eqs. (6)–(7) are discretized versions of Eq. (8), but the compartment-based model in (3) breaks down as $h\rightarrow 0$ as discussed in Section 2.2 of Engblom et al. (2009). The SPDE in Eq. (8) is consistent to the ones in the previous work (see Equation 1 in Kim et al. 2017 and Equation 3.24 in Dogan and Allen 2011). For more details, see derivations of the SPDE for diffusion in Section 3.1 of Dogan and Allen (2011) and the general version (Equations 8.2.54–8.2.56) in Sections 8.1-8.2 of Gardiner (2004).

## Multiscale Algorithms Combining Compartment-Based Models with SPDEs

In this section, we present a multiscale approach which uses both SPDEs and Markov chain models. We develop two algorithms, denoted **Scheme 1** and **Scheme 2** in what follows, which are applied to illustrative examples in Sects. 4, 5 and 6. Considering the same set up as in Sect. 2, we study a system of *N* chemically reacting species $$S_1,$$$$S_2$$, ..., $$S_N$$, which are diffusing (with diffusion constants $$D_i$$, $$i=1,2,\ldots ,N$$) in an elongated domain $$\varOmega = [0,L] \times [0,h_y] \times [0,h_z]$$, where $$L=Kh$$, given in Fig. 1. The domain $$\varOmega $$ is divided into *K* compartments (rectangular cuboids) with $$C_k = [(k-1)h,kh] \times [0,h_y] \times [0,h_z]$$ for $$k=1,2,\ldots , K$$.

The main goal of this paper is to replace the Markov chain description in a part of the computational domain by the corresponding SPDEs. Let us consider that we use the SPDE in Eq. (8) in the domain $\varOmega _s = [0,I] \times [0,h_y] \times [0,h_z]$ where $I = K_s h$ and $K>K_s\in {\mathbb {N}}$; i.e. we consider that the first $K_s$ compartments are described by a suitable discretization of the SPDE in Eq. (8) (see Fig. 1b). We only use the Markov chain model for the remaining $K_m=K-K_s$ compartments, i.e. in subdomain $\varOmega _m=[I,L] \times [0,h_y] \times [0,h_z]$. In this section, we develop an appropriate boundary condition on the interface *I* between $\varOmega _s$ and $\varOmega _m$.

In order to design the numerical scheme, the SPDE in Eq. (8) needs to be appropriately discretized. We denote by $\varDelta x$ the mesh size used in the discretization of the SPDE. There are two important cases: (i) $\varDelta x>h$ and (ii) $\varDelta x\le h$. In this section, we focus on case (ii), because we are interested in coupling the SPDE in Eq. (8) with Markov chain models. The case (i) is important when one uses discretized SPDEs to design efficient multiscale schemes, but this introduces additional discretization errors. We will discuss case (i) in Sect. 7. In $\varOmega _s$, each compartment of size *h* is discretized into $\alpha$ grid points ($\alpha \in {\mathbb {N}}$) with each grid size equal to $\varDelta x$. In the remaining part of the computational domain $\varOmega _m$, the compartment-based model is used. The state of the system of the multiscale model is described by vectors ${{\mathbf {X}}}^k(t)$, $k=1,2,\ldots ,K_s\alpha +K_m$. The vector ${{\mathbf {X}}}^k(t)$ for $k=1,2,\ldots ,K_s\alpha$ represents species ‘numbers’ in the mesh interval $[(k-1)\varDelta x,k\varDelta x]$ in the SPDE region $\varOmega _s$, i.e. it is related to spatio-temporal concentration ${{\mathbf {c}}}(x,t)$ used in the SPDE description by

$$\begin{aligned} {{\mathbf {X}}}^k(t) \approx h_y h_z \int _{(k-1)\varDelta x}^{k\varDelta x}{{\mathbf {c}}}(x,t) \,\mathrm {d}x. \end{aligned}$$

The vector ${{\mathbf {X}}}^{K_s\alpha +k}(t)$ for $k=1,2,\ldots , K_m$ represents species numbers in $C_{K_s+k}=[I+(k-1)h,I+kh]$ in the Markov chain region $\varOmega _m$, i.e. it is related to the state vector ${{\mathbf {Z}}}^k(t)$ used in the Markov chain description by ${{\mathbf {X}}}^{K_s\alpha +k}(t)={{\mathbf {Z}}}^{K_s+k}(t).$

**Fig. 2:**
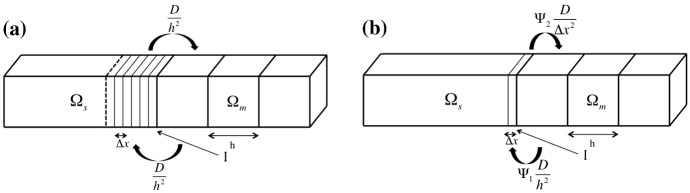
Schematic diagrams of **a** Scheme 1 and **b** Scheme 2 describing molecular transfer between $\varOmega _s$ and $\varOmega _m$. Note that the size of a virtual compartment in $\varOmega _s$ is *h* in panel (**a**)

We consider two different schemes to describe transfer of molecules near the interface *I* coupling discretized SPDEs and the Markov chain model, as shown in Fig. 2. Without loss of generality, both schemes are introduced for diffusion, because the description of reactions does not influence the transfer of molecules across the interface *I*. In **Scheme 1**, we assume that there is a virtual compartment, $C_{K_s}=[I-h,I]$, in $\varOmega _s$, where the molecules are partially treated using a compartment-based approach. Such overlap (handshaking) regions are common in many multiscale methodologies, including coupling molecular dynamics with Brownian dynamics simulations (Erban 2014, 2016), Brownian dynamics with PDEs (Franz et al. 2013), or in atomistic to continuum coupling methods (Miller and Tadmor 2009). We define a state vector

$$\begin{aligned} {{\mathbf {X}}}(t) = \left( {{\mathbf {X}}}^1(t)^{\mathrm {T}},{{\mathbf {X}}}^2(t)^{\mathrm {T}}, \ldots ,{{\mathbf {X}}}^{K_s\alpha +K_m}(t)^{\mathrm {T}}\right) ^{\mathrm {T}}, \end{aligned}$$

which is a $(K_s\alpha +K_m)N$-dimensional column vector. **Scheme 1** is described using the following evolution equation for state vector ${{\mathbf {X}}}(t)$:

9 $$\begin{aligned} {{\mathbf {X}}}(t)= & {} {{\mathbf {X}}}(0) + \sum _{k=2}^{K_s\alpha } \sum _{i=1}^N \Bigg \{ \int _0^t \frac{D_i}{\varDelta x^2} \left( X_i^k(s)-X_i^{k-1}(s)\right) \,\text{ d }s\nonumber \\&\qquad \qquad \qquad \qquad + \int _0^t \sqrt{\frac{D_i}{\varDelta x^2}\left( X_i^{k}(s)+X_i^{k-1}(s)\right) {\chi }_{\left\{ X_i^k(s)+X_i^{k-1}(s)\ge 0\right\} }} \, \text{ d }W_i^{k-1}(s) \Bigg \} \,{\varvec{\nu }}_{-,i}^k \nonumber \\&+ \sum _{k=K_s\alpha +2}^{K} \sum _{i=1}^N Y^k_{-,i} \left( \int _0^t \frac{D_i}{h^2} \, X_i^k(s) \, \text{ d }s \right) {\varvec{\nu }}_{-,i}^{k} \nonumber \\&+ \sum _{k=K_s\alpha +1}^{K-1} \sum _{i=1}^N Y^{k}_{+,i} \left( \int _0^t \frac{D_i}{h^2} \, X_i^{k}(s)\, \text{ d }s \right) {\varvec{\nu }}_{+,i}^{k} \nonumber \\&+ \underbrace{ \sum _{\ell =1}^{\alpha } \sum _{i=1}^N Y^{K_s\alpha +1}_{-,i} \left( \int _0^t \frac{D_i}{h^2} \, X_i^{K_s\alpha +1}(s)\, \text{ d }s \right) {\mathscr {I}}_{-,i}^{\ell }(t) \, {\varvec{\eta }}_{-,i}^{\ell } }_{\text {Markov chain }\rightarrow \text { SPDE}} \nonumber \\&+ \underbrace{ \sum _{\ell =1}^{\alpha } \sum _{i=1}^N Y^{K_s\alpha }_{+,i} \left( \int _0^t \frac{D_i}{h^2} \sum _{j=1}^{\alpha } X_i^{(K_s-1)\alpha +j}(s) {\chi }_{\left\{ \sum _{j=1}^{\alpha } X_i^{(K_s-1)\alpha +j}(s)\ge 1\right\} } \, \text{ d }s \right) {\mathscr {I}}_{+,i}^{\ell }(t) \, {\varvec{\eta }}_{+,i}^{\ell } }_{\text {SPDE }\rightarrow \text { Markov chain}},\nonumber \\ \end{aligned}$$

where the first sum on the right-hand side represents diffusion in $\varOmega _s$ (compare with Eq. (5) replacing *h* by $\varDelta x$). Note that the indicator function ${\chi }_{\left\{ X_i^k(s)+X_i^{k-1}(s)\ge 0\right\} }$ is used to make sure the term inside the square root not being negative. Here the symbols ${\varvec{\nu }}_{\pm ,i}^k$ describe $(K_s\alpha +K_m)N$-dimensional stoichiometric vectors. The second and third sums represent diffusion in the compartment-based region, $\varOmega _m$, where $Y_{\pm ,i}^k$ are independent unit Poisson processes (compare with Eq. (2)). The last two sums represent transition of a molecule from $\varOmega _m$ to $\varOmega _s$ and from $\varOmega _s$ to $\varOmega _m$, respectively. A molecule in $\varOmega _m$ in the boundary compartment, $C_{K_s+1}$, jumps to the SPDE domain with a rate $D_i/h^2$. A molecule which jumps is placed to one of the mesh points in the overlap compartment, $C_{K_s}$. To describe this process in Eq. (9), we have defined indicator functions

$$\begin{aligned} {\mathscr {I}}_{\pm ,i}^{\ell }(t) = {\chi }_{\left\{ U_{\pm ,i}(t)\in [I-h+(\ell -1)\varDelta x,I-h+\ell \varDelta x] \right\} }, \qquad \text{ for } \quad \ell =1,2,\ldots ,\alpha , \end{aligned}$$

where $U_{\pm ,i}(t)$ are independent random variables uniformly distributed on interval $[I-h,I]$ for each *t* and *i*. Stoichiometric vectors, ${\varvec{\eta }}_{\pm ,i}^{\ell }$ for $\ell =1,2,\ldots ,\alpha$, $i=1,2,\ldots ,N$, give changes due to the diffusion of the *i*th species between the $\ell$th SPDE discretization point in $C_{K_s}$ and the compartment $C_{K_s+1}$ across the interface *I*. Transition of a molecule from $\varOmega _s$ to $\varOmega _m$ is described by the last term of Eq. (9) using time-changed Poisson processes. A molecule, anywhere in the overlap compartment $C_{K_s}$, can be transferred with a rate $D_i/h^2$. The corresponding molecule is then randomly subtracted from one of $\alpha$ discretization grid points which are in $C_{K_s}$. Note that the molecular copy number, $\sum _{j=1}^{\alpha } X_i^{(K_s-1)\alpha +j}(s)$, in the last term of Eq. (9) can be non-integer value due to the non-integer concentration in $C_{K_s}$. To prevent $X_i^{(K_s-1)\alpha +j}(s)$ being negative due to the molecular transfer from $\varOmega _s$ to $\varOmega _m$, another indicator function is used in the last term of Eq. (9) to set the propensity as zero if the total molecular copy number in $C_{K_s}$ is less than 1.

**Scheme 2** is described in terms of two unknown parameters, denoted $\varPsi _1$ and $\varPsi _2$, by the following evolution equation for the state vector ${{\mathbf {X}}}(t)$:

10 $$\begin{aligned} {{\mathbf {X}}}(t)= & {} {{\mathbf {X}}}(0) + \sum _{k=2}^{K_s\alpha } \sum _{i=1}^N \Bigg \{ \int _0^t \frac{D_i}{\varDelta x^2} \left( X_i^k(s)-X_i^{k-1}(s)\right) \,\text{ d }s\nonumber \\&\qquad \qquad \qquad \qquad + \int _0^t \sqrt{\frac{D_i}{\varDelta x^2}\left( X_i^{k}(s)+X_i^{k-1}(s)\right) {\chi }_{\left\{ X_i^k(s)+X_i^{k-1}(s)\ge 0\right\} }} \, \text{ d }W_i^{k-1}(s) \Bigg \} \,{\varvec{\nu }}_{-,i}^k \nonumber \\&+ \sum _{k=K_s\alpha +2}^{K} \sum _{i=1}^N Y^k_{-,i} \left( \int _0^t \frac{D_i}{h^2} \, X_i^k(s)\, \text{ d }s \right) {\varvec{\nu }}_{-,i}^{k} \nonumber \\&+ \sum _{k=K_s\alpha +1}^{K-1} \sum _{i=1}^N Y^{k}_{+,i} \left( \int _0^t \frac{D_i}{h^2} \, X_i^{k}(s)\, \text{ d }s \right) {\varvec{\nu }}_{+,i}^{k} \nonumber \\&+ \underbrace{ \sum _{i=1}^N Y^{K_s\alpha +1}_{-,i} \left( \int _0^t \varPsi _1 \frac{D_i}{h^2} \, X_i^{K_s\alpha +1}(s)\,\text{ d }s \right) {\varvec{\nu }}_{-,i}^{K_s\alpha +1}}_{\text {Markov chain }\rightarrow \text { SPDE}} \nonumber \\&+ \underbrace{ \sum _{i=1}^N Y^{K_s\alpha }_{+,i} \left( \int _0^t \varPsi _2 \frac{D_i}{\varDelta x^2} \, X_i^{K_s\alpha }(s) {\chi }_{\left\{ X_i^{K_s\alpha }(s)\ge 1 \right\} } \, \text{ d }s \right) {\varvec{\nu }}_{+,i}^{K_s\alpha } }_{\text {SPDE }\rightarrow \text { Markov chain}}. \end{aligned}$$

The first three sums on the right hand side in Eq. (10) are identical to those in Eq. (9). The fourth and fifth sums describe molecular transfer between the last grid point in $\varOmega _s$ and the boundary compartment $C_{K_s+1}$. A molecule in $\varOmega _m$ in the boundary compartment, $C_{K_s+1}$, jumps to the last grid point of the SPDE domain with rate $\varPsi _1 D_i/h^2$, and the transfer rate in the opposite direction is $\varPsi _2 D_i/\varDelta x^2$. Note that $X_i^{K_s\alpha }(s)$ in the fifth term of Eq. (10) can be non-integer value due to the non-integer concentration in $\varOmega _s$. To prevent $X_i^{K_s\alpha }(s)$ being negative due to the molecular transfer from $\varOmega _s$ to $\varOmega _m$, we use an indicator function to set the propensity as zero if the molecular copy number in the last grid point in $\varOmega _s$ is less than 1.

**Table 1:** Pseudocode for the multiscale reaction–diffusion algorithm with Scheme 1 applied to simulation of diffusion

**[A]**	Set $t=0$ and $t_s=\varDelta t$. Initialize species numbers, ${{\mathbf {X}}}(0)$, in $\varOmega _m$ and $\varOmega _s$. Then, generate random numbers $r_1$ and $r_2$ uniformly distributed in (0, 1). Set $\tau$ so that $\tau =- a_0^{-1} \log \left( r_1\right)$, where $a_0$ is defined in Eq. (11). Set the next time when the diffusion occurs in $\varOmega _m$ as $t_m=\tau$
**[B]**	If $t_m\le t_s$
	$\bullet$ Set $t=t_m$
	$\bullet$ Use $r_2$ to determine which diffusive jump occurs. Each diffusive jump to the left (resp. to the right) has the probability $a_{-,i}^k/a_0$ (resp. $a_{+,i}^k/a_0$) to occur
	$\bullet$ If the selected diffusive jump only includes internal compartments in $\varOmega _m$, update species numbers in the corresponding compartments
	$\bullet$ If the diffusion occurs across the interface from $\varOmega _m$ to $\varOmega _s$, update the species number in $C_{K_s}$ by transferring one molecules from $C_{K_s+1}$ to the corresponding grid point in $\varOmega _s$
	$\bullet$ If the diffusion occurs across the interface from $\varOmega _s$ to $\varOmega _m$, update the species number in $C_{K_s+1}$ by adding one molecule and subtracting one from the corresponding grid point in $\varOmega _s$
	$\bullet$ Generate random numbers $r_1$ and $r_2$ uniformly distributed in (0, 1). Set $\tau$ so that $\tau =- a_0^{-1} \log \left( r_1\right)$, where $a_0$ is defined in Eq. (11). Set the next time when the diffusion occurs in $\varOmega _m$ as $t_m=t+\tau$
**[C]**	If $t_s\le t_m$
	$\bullet$ Set $t=t_s$
	$\bullet$ Use Eq. (12) to update the SPDE part of the system from *t* to $t+\varDelta t$
	$\bullet$ Set the next time of the update of the SPDE part as $t_s=t+\varDelta t$
**[D]**	Repeat steps **[B]**–**[C]** until the simulation ends

To determine parameters $\varPsi _1$ and $\varPsi _2$ of **Scheme 2**, we use the discretization of the PDE for diffusion using a finite volume approximation (Bernstein 2005). It gives the jump coefficient of the *i*th species from the *j*th compartment to the neighbouring $j'$th compartment as $D_i/(h_{j}|a_{j}-a_{j'}|)$, where $h_j$ is the length of the *j*th compartment and $a_j$ and $a_{j'}$ are the centres of the *j*th and $j'$th compartments, respectively. Considering the size of the domain allowed for molecule transfer across the interface in **Scheme 2**, we set $|a_j-a_{j'}|=(\varDelta x+h)/2$. We take $h_j=\varDelta x$ for the jump coefficient from $\varOmega _s$ to $\varOmega _m$ and $h_j=h$ for the jump coefficient from $\varOmega _m$ to $\varOmega _s$. Then, we match the jump coefficients to the rate constants for jump across the interface given in Eq. (10) to derive the following formula for the parameters of **Scheme 2**

$$\begin{aligned} \varPsi _1 = \frac{2h}{\varDelta x+h} \qquad \text{ and } \qquad \varPsi _2 = \frac{2 \varDelta x}{\varDelta x+h}. \end{aligned}$$

The multiscale algorithm for **Scheme 1** for the case of diffusion only is given in Table 1. We denote a propensity of diffusion of the *i*th species in the $(K_s+k)$th compartment in $\varOmega _m$ to the left (resp. right) as $a_{-,i}^k(t) = D_i/h^2 X_i^{K_s\alpha +k}(t)$, for $k=1,2,\ldots ,K_m$, (resp. $a_{+,i}^k(t) = D_i/h^2 X_i^{K_s\alpha +k}(t)$, for $k=1,2,\ldots ,K_m-1$) for $i=1,2,\ldots , N$. This definition also includes the propensity of a diffusive jump of the *i*th species from the Markov chain domain, given as $a_{-,i}^1(t).$ We denote a propensity of diffusive jump of the *i*th species from the SPDE domain by

$$\begin{aligned} a_{+,i}^0(t) = \frac{D_i}{h^2} \sum _{j=1}^\alpha X_i^{(K_s-1)\alpha +j}(t). \end{aligned}$$

Then, we define total propensity in $\varOmega _m$

11 $$\begin{aligned} a_0= & {} \sum _{i=1}^N \sum _{k=1}^{K_m} a_{-,i}^k +\sum _{i=1}^N \sum _{k=0}^{K_m-1} a_{+,i}^k . \end{aligned}$$

Total propensity $a_0$ is used in steps **[A]** and **[B]** in the pseudocode in Table 1 to select time when the next event occurs in $\varOmega _m$. The pseudocode denotes the time of the next update in each subdomain as $t_s$ and $t_m$, and the current time as *t*. In step **[B]**, we update the compartment-based part of the system. In step **[C]**, we update the SPDE part of the system by

12 $$\begin{aligned} {{\mathbf {X}}}(t+\varDelta t)= & {} {{\mathbf {X}}}(t) + \sum _{k=2}^{K_s\alpha } \sum _{i=1}^N \left\{ \frac{D_i}{\varDelta x^2} \left( X_i^k(t)-X_i^{k-1}(t)\right) \varDelta t \right. \nonumber \\&\left. +\, \sqrt{\frac{D_i \varDelta t}{\varDelta x^2}\big (X_i^k(t)+X_i^{k-1}(t)\big ) \chi _{\big \{X_i^k(t)+X_i^{k-1}(t)\ge 0\big \}}} \, \zeta _i^{k-1} \right\} \,{\varvec{\nu }}_{-,i}^k , \end{aligned}$$

where $\zeta _i^{k-1}$ are independent normally distributed random numbers with zero mean and unit variance.

## Application: Static Boundary

In this section, we apply the multiscale approach to examples in which we know a priori the position of the boundary *I* between $\varOmega _s$ and $\varOmega _m$. Generalization to a more complicated case with a moving boundary is presented in Sect. 5.

### A Morphogen Gradient Model

We consider a morphogen gradient model in $\varOmega = [0,L]\times [0,h_y]\times [0,h_z]$. It consists of one chemical species *S*, i.e. $Z^k(t)$ is a scalar describing the number of molecules of *S* in $C_k$. The state of the Markov chain model is described by the *K*-dimensional column vector ${{\mathbf {Z}}}(t)=\left( Z^1(t),Z^2(t),\ldots ,Z^K(t)\right) ^{\mathrm {T}}$. Morphogen is subject to diffusion which is described by Eq. (2). There are also two reactions in the system. Morphogen, *S*, is produced in the first compartment with rate *J*, i.e. the propensity is $\lambda _1^1\left( Z^1\right) =J$. Morphogen degrades everywhere with rate $\delta$, i.e. with propensity $\lambda _2^k\left( Z^k\right) =\delta Z^k$ for $k=1,2,\ldots ,K$. In all stochastic simulations of the morphogen gradient model, we assume that 500 morphogen molecules are initially uniformly distributed in the half of the domain, $\varOmega _s=[0,L/2]\times [0,h_y]\times [0,h_z]$. The parameters are given in Table 2.

**Table 2:** Parameter values in the morphogen gradient model studied in Sect. 4.1

Notations	Description	Values
*L*	Length of the domain	$20\,\upmu \hbox {m}$
*D*	Diffusion coefficient	$0.8\,\upmu \hbox {m}^2\,\hbox {s}^{-1}$
$\delta$	Degradation rate	$0.05\,\hbox {s}^{-1}$
*J*	Production rate	$25\,\mu \hbox {m}^{-2}\,\hbox {s}^{-1}$
$\varDelta x$	Spatial discretization in $\varOmega _s$	$0.2\,\upmu \hbox {m}$
*h*	Spatial discretization in $\varOmega _m$	$1\,\upmu \hbox {m}$
$h_y$, $h_z$	Spatial discretization in *y* and *z* directions	$1\,\upmu \hbox {m}$
$\varDelta t$	Time discretization for SPDE	$5 \times 10^{-4}~\hbox {s}$

**Fig. 3:**
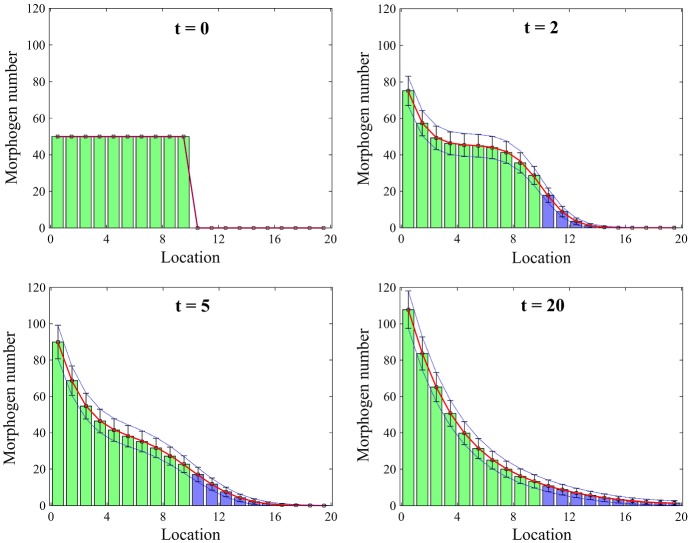
Comparison between mean numbers of morphogens and their standard deviations from the mean using the analytic solution (red lines and blue dotted lines) and Scheme 1 (green bars and blue bars for the means in $\varOmega _s$ and $\varOmega _m$, respectively, and error bars for the standard deviations) (Color figure online)

Denoting *c*(*x*, *t*) the morphogen density at point *x* and time *t*, the deterministic model can be written as PDE

$$\begin{aligned}&\frac{\partial c}{\partial t} = D \frac{\partial ^2 c}{\partial x^2} - \delta \, c, \qquad \text{ with } \text{ boundary } \text{ conditions } \qquad \\&\quad \left. -D\frac{\partial c}{\partial x}\right| _{x=0} = J, \quad \left. D\frac{\partial c}{\partial x}\right| _{x=L} = 0, \end{aligned}$$

where *D* is the diffusion constant of morphogen *S*. We apply the multiscale approach using both schemes developed in Sect. 3. Since the morphogen is produced at the left end, the morphogen has a decreasing gradient as it goes towards *L*. Therefore, we split the spatial domain in half and set the left half as $\varOmega _s$ and the right half as $\varOmega _m$, i.e. $I=L/2$. The $(K_s\alpha +K_m)$-dimensional state vector of the multiscale model is denoted ${{\mathbf {X}}}(t)=\left( X^1(t),X^2(t),\ldots ,X^{K_s\alpha +K_m}(t)\right) ^{\mathrm {T}}$. Note that the morphogen molecules are only produced in the first discretization mesh point with size $\varDelta x$ in $\varOmega _s$. In Fig. 3, we simulate the morphogen gradient model using **Scheme 1** of the multiscale algorithm. We calculate $10^4$ realizations of the sample paths of the stochastic process and present mean and standard deviations of the morphogen numbers in $\varOmega$ at different times, $t=0,2,5,20\,\hbox {s}$. Morphogen numbers in $\alpha$ grid points of $\varOmega _s$ are summed so that they can be compared to the numbers in the underlying Markov chain model. We compare the results with mean and standard deviations of the morphogen numbers which we calculate analytically using matrix analysis for reaction–diffusion Markov chain models (Gadgil et al. 2005; Kang et al. 2012b). In Fig. 3, morphogen numbers in $\varOmega _s$ (resp. in $\varOmega _m$) are expressed as green bars (resp. blue bars). Error bars represent one standard deviations from the mean number of morphogens in each compartment. Mean and standard deviations of the morphogen numbers from the analytic solution are drawn as a red line and blue dotted lines. The results using the multiscale algorithm match perfectly to the ones from the exact solution.

**Fig. 4:**
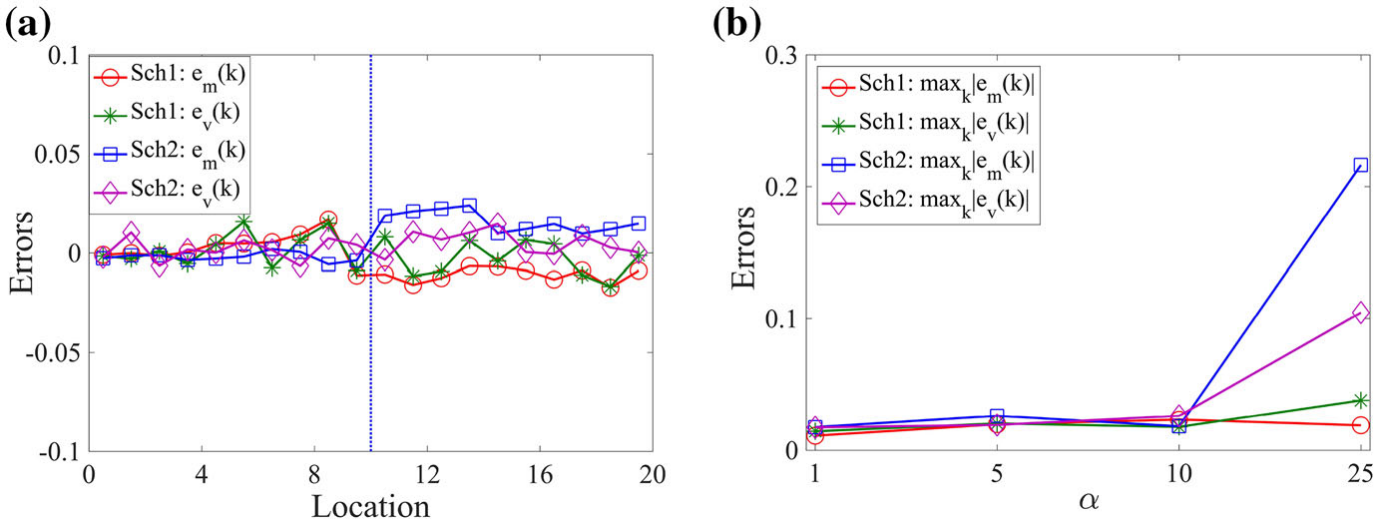
**a** Errors $e_m(k)$ and $e_v(k)$ given by Eq. (13) are computed at time $50\,\mathrm {s}$. **b** The maximum absolute values of the errors $e_m(k)$ and $e_v(k)$ given by Eq. (13) are computed at time $50\,\mathrm {s}$ with a static boundary and different values of $\alpha$. The maximum value of the errors is taken over all region, $\varOmega$. Red and green lines are relative errors of the means and standard deviations between the analytic solution of the Markov chain model and Scheme 1. Blue and purple lines are relative errors between the Markov chain model and Scheme 2 (Color figure online)

In Fig. 4, we present relative errors of the means and standard deviations of the number of molecules between the Markov chain model and multiscale model. The analytic solution is used for the statistics of the Markov chain model, and both schemes are applied numerically for the multiscale approaches. Errors are defined as

13 $$\begin{aligned} \begin{aligned} \displaystyle e_m(k)&= \left\{ \begin{array}{ll} \displaystyle 1 - \frac{\text{ E } \left[ \sum _{\ell =1}^{\alpha } X^{(k-1)\alpha +\ell } \right] }{\text{ E } \left[ Z^k \right] },&{}\displaystyle \text {for } k=1,2,\ldots ,\frac{K}{2}, \\ \displaystyle 1 - \frac{\text{ E }\left[ X^{k+K(\alpha -1)/2} \right] }{\text{ E }\left[ Z^k\right] },&{}\displaystyle \text {for } k=\frac{K}{2}+1,\ldots ,K, \end{array} \right. \\ e_v(k)&= \left\{ \begin{array}{ll} \displaystyle 1 - \frac{\sigma \left[ \sum _{\ell =1}^{\alpha } X^{(k-1)\alpha +\ell } \right] }{\sigma \left[ Z^k\right] },\; &{} \displaystyle \text {for} \;k=1,2,\ldots ,\frac{K}{2},\\ \displaystyle 1 - \frac{\sigma \left[ X^{k+K(\alpha -1)/2} \right] }{\sigma \left[ Z^k\right] }, &{} \displaystyle \text {for} \; k=\frac{K}{2}+1,\ldots ,K, \end{array} \right. \end{aligned} \end{aligned}$$

where $\text{ E }[\cdot ]$ and $\sigma [\cdot ]$ represent a mean and standard deviation. In Fig. 4a, red and green lines represent $e_m(k)$ and $e_v(k)$ at time $t=50\,\hbox {s}$ using **Scheme 1**, respectively, and blue and purple lines are for **Scheme 2**. We observe that the relative errors in Eq. (13) are less than $4\,\%$ in the entire simulation domain. In Fig. 4b, we compare the maximum absolute values of the relative errors defined in Eq. (13) with $\alpha =1,\,5,\,10,\,25$ and fixed compartment size *h* where $\alpha =h/\varDelta x$. In both schemes, the relative errors are in a range of less than $4\,\%$ except for the case when $\alpha =25$ with **Scheme 2**. The relative errors in the mean and standard deviation become significantly larger when we apply the multiscale algorithm using **Scheme 2** with $\alpha =25$. In this case, the mean in $C_{K_s+1}$ gets larger than the mean in $C_{K_s}$ which shows a bias in the method for larger values of $\alpha$ (the exact mean number of molecules decreases along the *x*-axis). We provide an explanation of this phenomenon in the next section.

### A Diffusion Model with Two Compartments

In Sect. 4.1, we have observed that the error of **Scheme 2** increases when we decrease the ratio of the numerical discretization in $\varOmega _s$ and the compartment size. When $\alpha =25$, the mean number of molecules of the morphogen does not have a decreasing gradient across the interface *I* in **Scheme 2**. To investigate this numerical error, we consider diffusion in a theoretical two-compartment model. It is similar to the one in Sect. 4.1, but we set $J=\delta =0$ so that there is no flux or degradation of the morphogen. We use $L=2h$ and $I=h$ ($=1\,\mu \hbox {m}$) so that $\varOmega _s=[0,h]\times [0,h_y]\times [0,h_z]$ and $\varOmega _m=[h,2h]\times [0,h_y]\times [0,h_z]$. Then, each region consists of one compartment, $K_s=K_m=1$, and ${{\mathbf {X}}}(t)$ is an $(\alpha +1)$-dimensional vector.

**Fig. 5:**
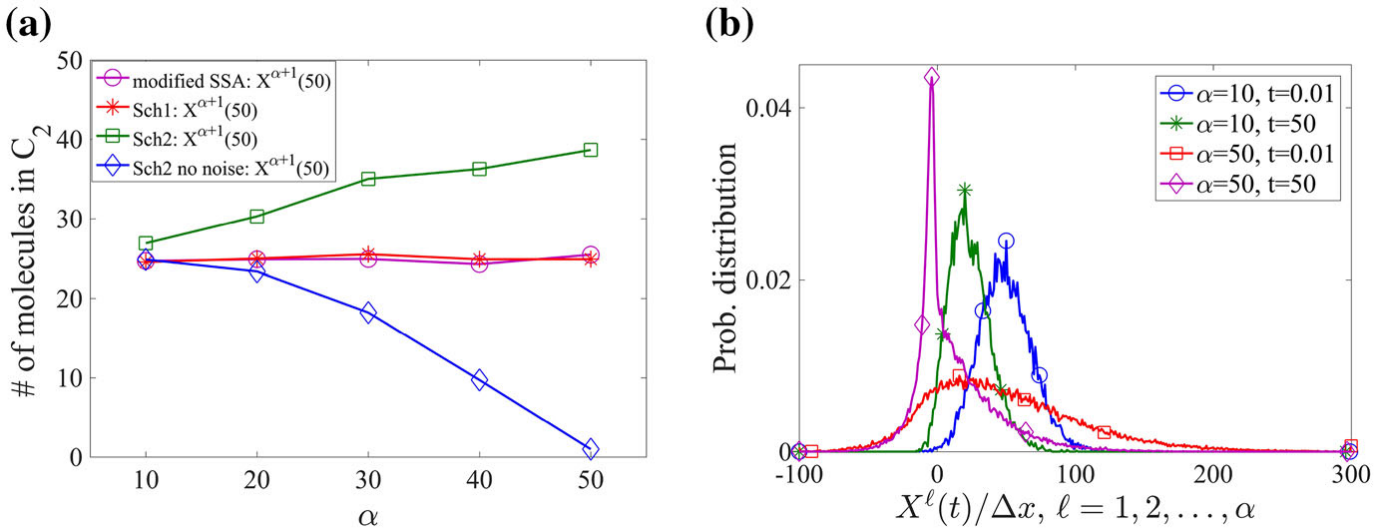
**a** The mean number of morphogens in $C_{2}$ at time $t=50\,\mathrm {s}$. Different simulation methods are compared with $\alpha =10$, 20, 30, 40, 50: the Gillespie SSA with multigrid discretization ($\alpha$ grid points with size $\varDelta x$ and one grid point with size *h*), Scheme 1, Scheme 2 and “Scheme 2 with no noise” due to diffusion in $\varOmega _s$. **b** The probability distribution of the normalized morphogen number in $C_1$ with Scheme 2. The probability distributions are computed for $X^{\ell }(t)/\varDelta x$, $\ell =1,2,\ldots ,\alpha$ and compared among the cases with $\alpha =10$, 50 at time $t=0.01$, $50\,\mathrm {s}$. Initially, 50 molecules are located in $\varOmega _s$ in panels (**a**, **b**) (Color figure online)

In Fig. 5a, we present simulation results of the two-compartment model using **Scheme 1** (red line) and **Scheme 2** (green line) with $\alpha =10,$ 20, 30, 40, 50 and compare them to the simulation result of the Markov chain model using the Gillespie SSA (purple line). The Markov chain model has $\alpha +1$ numerical grid points where the first $\alpha$ ones are with size $\varDelta x=h/\alpha$ and the last one with size *h*. Diffusion of molecules is simulated by jumps from grid points to their nearest neighbours, i.e. the numerical meshes in the Markov chain model are coupled by diffusion in the same way as it is done in **Scheme 2**. Applying both multiscale algorithms and the Gillespie SSA, we compare the mean morphogen numbers in the second compartment computed from 100 realizations of simulation. Using 50 molecules in total, the exact value of the mean numbers of molecules in $C_k$, $k=1,2$, is 25. Notice that **Scheme 1** and the Gillespie SSA with two mesh sizes correctly approximate the means. However, **Scheme 2** overestimates the mean morphogen number in $C_2$ as $\alpha$ gets large. To understand where the numerical error arises, we also simulate **Scheme 2** without the noise term in the SPDEs (marked as a blue line in Fig. 5a), i.e. we remove the term with a square root in Eq. (10).

In Fig. 5a, we observe that the mean morphogen number in $C_2$, $E[X^{\alpha +1}]$, is underestimated as $\alpha$ increases when we use **Scheme 2** without noise term in the SPDEs. Note that $X^{\ell }$, $\ell =1,2,\ldots ,\alpha$, always have non-negative integer values due to no noise term in modified Eq. (10). The molecular transfer from $\varOmega _s$ to $\varOmega _m$ occurs when $X^{\alpha } \ge 1$. However, the frequency of this transfer is not sufficient as $\alpha$ gets large, which lowers $X^{\alpha +1}$. On the other hand, with noise terms included in Eqs. (9) and (10), there are more chances that $X^{\ell }<0$ for some $\ell =1,2,\ldots ,\alpha$ due to large fluctuations with a small number of molecules as $\alpha$ gets large. Then, it is more frequent that $X^{\alpha }\ge 1$ due to the fact that $\sum _{\ell =1}^{\alpha +1}X^{\ell }=50$ and $X^{\ell }<0$ for some $\ell =1,2,\ldots ,\alpha$. More frequent molecular transfer from $\varOmega _s$ to $\varOmega _m$ causes overestimation of the mean morphogen number in $C_2$ in **Scheme 2**.

In Fig. 5b, we compare distributions of the morphogen numbers when $\alpha =10$ and 50. The distributions are computed from 1000 realizations of simulation when $t=0.01\,\mathrm {s}$ and $50\,\mathrm {s}$. Each distribution is computed over all $X^{\ell }$, $\ell =1,2,\ldots ,\alpha$ so that we can display an overall range of the morphogen number in each discretization of $\varOmega _s$. Each $X^{\ell }$ is normalized by $\varDelta x$ so that the distributions can be compared for different $\alpha$’s. The normalized mean morphogen number (density) in $\varOmega _s$ decreases significantly in both cases with $\alpha =10$ and 50 as time evolves. On the other hand, the variance of the morphogen density is much greater for $\alpha =50$ than for $\alpha =10$ at $t=0.01\,\mathrm {s}$ due to the lower morphogen number in each discretization of $\varOmega _s$. Therefore, we conclude that the error in **Scheme 2** strongly depends on the size of fluctuations close to the interface. On the other hand, the molecular transfer from $\varOmega _s$ to $\varOmega _m$ is decided by $\sum _{\ell =1}^{\alpha }X^{\ell }$ in **Scheme 1**. This makes **Scheme 1** more robust than **Scheme 2** for large values of $\alpha$.

## Application: Moving Boundary

In some applications (Robinson et al. 2014), it is difficult to decide a position of the interface *I* a priori. In this section, we extend the presented algorithm to the case when the location of the interface *I*(*t*) between $\varOmega _s$ and $\varOmega _m$ moves in time, based on the number of molecules in each part of the domain. The multiscale approach with the adaptive interface is applied to the example introduced in Sect. 4.

**Table 3:** Pseudocode for the adaptive multiscale reaction–diffusion algorithm with Scheme 1 applied to simulation of diffusion

**[A’]**	Set $t=0$, $t_s=\varDelta t$ and $k=1$. Initialize species numbers, ${{\mathbf {X}}}(0)$, in $\varOmega _m$ and $\varOmega _s$, and the boundary location, *I*(0). Then, generate random numbers $r_1$ and $r_2$ uniformly distributed in (0, 1). Set $\tau$ so that $\tau =- a_0^{-1} \log \left( r_1\right)$, where $a_0$ is defined in Eq. (11). Set the next time when the diffusion occurs in $\varOmega _m$ as $t_m=\tau$
**[B’]**	Repeat steps [B]–[C] of algorithm in Table 1 until time $k(n_c \varDelta t)$
**[C’]**	Update position of interface *I*(*t*), if necessary, by
	$\bullet$ If $\displaystyle \sum _{\ell =1}^{\alpha } X^{(K_s-1)\alpha +\ell }<Q_\mathrm{lower}$, set $I(t)=I(t-n_c\varDelta t)-h$
	$\bullet$ If $\displaystyle \sum _{\ell =1}^{\alpha } X^{(K_s-1)\alpha +\ell } \ge Q_\mathrm{lower}$ and $X^{K_s\alpha +1}>Q_\mathrm{upper}$, set $I(t)=I(t-n_c\varDelta t)+h$
	$\bullet$ Increase *k* by 1, i.e. $k = k + 1$
**[D’]**	Repeat steps **[B’]**–**[C’]** until the simulation ends

**Fig. 6:**
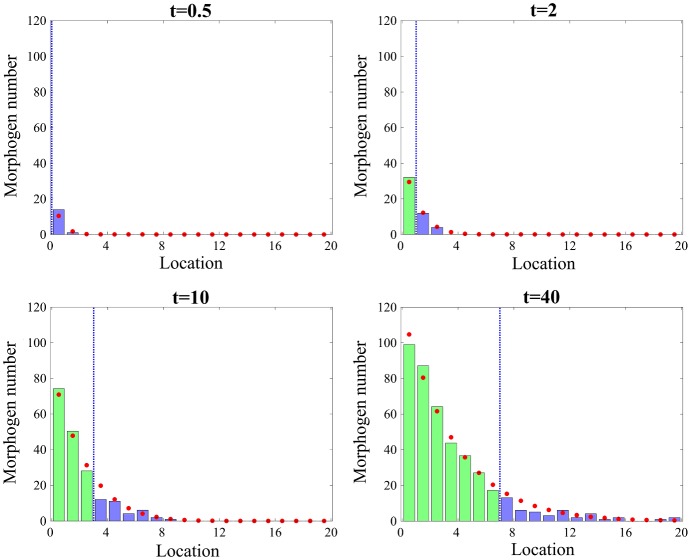
Comparison between one realization of the number of morphogens using Scheme 1 with a moving interface, given in Table 3 (green bars and blue bars for the morphogen numbers in $\varOmega _s$ and $\varOmega _m$, respectively) and the mean number of molecules given analytically by the compartment-based model (red dots). A blue dotted line represents the location of the interface *I*(*t*) (Color figure online)

The adaptive algorithm is described in Table 3. Following (Robinson et al. 2014), we introduce two thresholds denoted $Q_\mathrm{upper}$ and $Q_\mathrm{lower}$ ($Q_\mathrm{upper}\ge Q_\mathrm{lower}$), and one integer parameter $n_c$. We initialize the position of the interface $I(0)=0$ in step **[A’]**, i.e. we initially model the whole domain using the detailed compartment-based approach. We run the original **Scheme 1** until time $n_c\varDelta t$. We check whether the interface *I*(*t*) should be moved in step **[C’]**. If the number of molecules in the compartment next to the interface in $\varOmega _s$ is smaller than $Q_\mathrm{lower}$, a compartment-based model is used in that region. On the other hand, if the number of molecules in the boundary compartment next to interface *I*(*t*) in $\varOmega _m$ is larger than threshold $Q_\mathrm{upper}$, the corresponding compartment is transferred to the SPDE region where the molecules are redistributed uniformly in $\alpha$ grid points. Due to the uniform redistribution of the molecules, rapid changing of the interface *I*(*t*) introduces more errors. Note that in **Scheme 1** with a fixed boundary, one molecule has been chosen randomly from $\alpha$ discretizations of $C_{K_s}$ in $\varOmega _s$ and transferred to $C_{K_s+1}$ in $\varOmega _m$. Similarly, we have taken one molecule from $C_{K_s+1}$ and transferred the molecule to the randomly chosen SPDE mesh point in $C_{K_s}$. However, in the adaptive algorithm, we modify the setting of **Scheme 1** so that a molecule is taken uniformly from the entire region of $C_{K_s}$ and transferred to $C_{K_s+1}$, i.e. $1/\alpha$ molecule is subtracted in all $\alpha$ SPDE grid points of $C_{K_s}$. Similarly when the molecule is transferred from $C_{K_s+1}$ to $C_{K_s}$, $1/\alpha$ molecule is added in all $\alpha$ grid points of $C_{K_s}$. Without this modification of the setting in **Scheme 1**, the appropriate level of the morphogen gradient is not formed in Fig. 6.

The adaptive algorithm **[A’]**–**[D’]** is applied to the morphogen gradient model introduced in Sect. 4, and the results are presented in Fig. 6. We use $Q_\mathrm{lower}=15$, $Q_\mathrm{upper}=25$ and $n_c=10$. Other parameters are given in Table 2. Our initial condition is $X^k(0)=0$, for $k=1,2,\ldots , K_s\alpha +K_m$, i.e. the system starts with no molecules and the gradient is formed during the simulation. In Fig. 6, one realization of the algorithm in Table 3 at different times $t=0.5$, 2, 10, $40\,\hbox {s}$ is presented. The green and blue bars represent the numbers of molecules in the corresponding compartments in $\varOmega _s$ and $\varOmega _m$, respectively. The blue dotted line represents interface *I*(*t*) between two regions, and the red circles are the mean numbers of molecules obtained from the analytic solution of the stochastic model. Our results show that the boundary between two regions is moving to the right in time as the molecule numbers increase due to the production on the left.

**Fig. 7:**
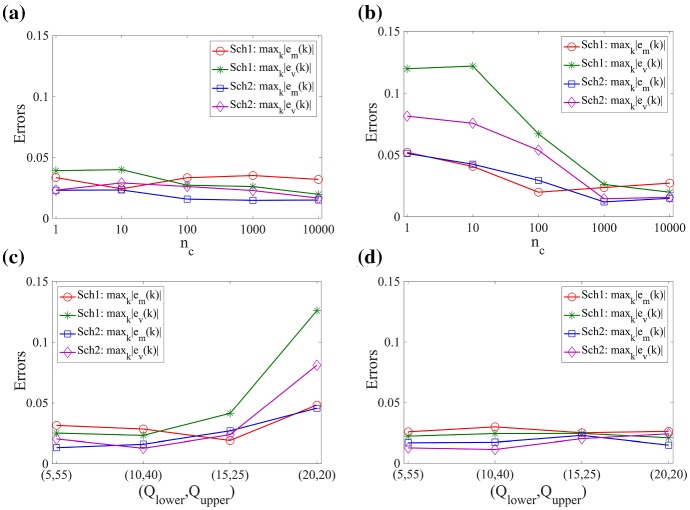
The maximum absolute values of the errors defined by Eq. (13) at time $50\,s$ using the multiscale algorithms, with a moving boundary. Different values of $n_c=1$, 10, $10^2$, $10^3$, $10^4$, are used with fixed thresholds **a**$(Q_\mathrm{lower},Q_\mathrm{upper})=(15,25)$ and **b**$(Q_\mathrm{lower},Q_\mathrm{upper})=(20,20)$. Different threshold values $(Q_\mathrm{lower},Q_\mathrm{upper})=(5,55)$, (10, 40), (15, 25), (20, 20) are used with, **c**$n_c=1$ and **d**$n_c=10^3$. Red and green lines are the maximal relative errors of the means and standard deviations between the analytic solution of the Markov chain model and Scheme 1, given by Eq. (13). Blue and Purple lines are maximal relative errors between the Markov chain model and Scheme 2, given by Eq. (13) (Color figure online)

In Fig. 7a and b, we simulate the adaptive algorithm with fixed thresholds for a range of values of $n_c=1$, 10, $10^2$, $10^3$, $10^4$, which are the numbers of time steps to check the criterion to move the interface *I*(*t*) in step **[C’]**. Two sets of fixed thresholds are chosen, $(Q_\mathrm{lower},Q_\mathrm{upper})=(15,25)$ in (a) and $(Q_\mathrm{lower},Q_\mathrm{upper})=(20,20)$ in (b). In Fig. 7c and d, we simulate the adaptive algorithm with fixed numbers of time steps, $n_c$, for different values of $Q_\mathrm{lower}$ and $Q_\mathrm{upper}$, which are the threshold values to check before we move the interface *I*(*t*) in step **[C’]**. We use the following pairs of the values for the thresholds: $(Q_\mathrm{lower},Q_\mathrm{upper})=(5,55)$, (10, 40), (15, 25), (20, 20). Two fixed numbers of time steps are used, $n_c=1$ in (c) and $n_c=1000$ in (d). As shown in Fig. 7b and c, we observe that the maximum absolute values of the relative errors increase as the number of time steps, $n_c$, or the size of the threshold window, $Q_\mathrm{upper}-Q_\mathrm{lower}$, gets smaller. This is because the small size of the number of time steps (or the threshold window) makes the interface location change frequently, which causes additional errors. On the other hand, Fig. 7a and d do not show similar pattern since large size of the threshold window ($Q_\mathrm{upper}-Q_\mathrm{lower}=10$) and the number of time steps ($n_c=10^3$) prevent frequent movement of the interface location. Overall, **Scheme 2** has slightly smaller errors than **Scheme 1**. In Fig. 7, the maximum absolute values of the relative errors are calculated using $10^4$ realizations of simulation using **Scheme 1** or **2** for each value of $n_c$ and for each set of values of $(Q_\mathrm{lower},Q_\mathrm{upper})$ and using the analytic solution of the Markov chain model.

## Applications: Multiple Species

In this section, we illustrate the applicability of the presented multiscale approaches to chemical systems with multiple species. Since different chemical species can have very different molecular distributions in the computational domain, the partition of the computational domain into subdomains $\varOmega _s$ and $\varOmega _m$ can be species dependent. We use the pom1p gradient model from Saunders et al. (2012) to illustrate a multiscale approach, where each species has a different partition into $\varOmega _s$ and $\varOmega _m$ depending on its molecular distribution. The model consists of two species, slow-diffusing pom1p clusters, denoted $S_1$, and fast-diffusing pom1p particles, denoted $S_2$. We use pseudo 1-dimensional domain $\varOmega$ as in Fig. 1, where $L=14\,\mu \hbox {m}$, which is divided into $K=40$ compartments, $C_k$, $k=1,2,\ldots ,K$. Both $S_1$ and $S_2$ are produced in the whole computational domain with space-dependent rates given by Saunders et al. (2012), i.e. with propensities

$$\begin{aligned} \lambda _j^k(\mathbf{Z }^k)= & {} a_j \exp {\left[ -a_6\left( k-\frac{K+1}{2}\right) ^2\right] } \end{aligned}$$

where $j=1,2$, $k=1,2,\ldots ,K$, and $a_1$, $a_2$ and $a_6$ are constants given in Table 4. In addition to production, species $S_1$ and $S_2$ are subject to the following reactions which take place in the whole domain

$$\begin{aligned} S_1 \longrightarrow S_2, \qquad S_1+S_2 \longrightarrow S_1+S_1, \qquad S_2 \longrightarrow \varnothing , \end{aligned}$$

with the corresponding propensities given by

$$\begin{aligned} \lambda _3^{k}({{\mathbf {Z}}}^{k}) = a_3 Z_1^k, \qquad \lambda _4^{k}({{\mathbf {Z}}}^{k}) = a_4 Z_1^k Z_2^k, \qquad \lambda _5^{k}({{\mathbf {Z}}}^{k}) = a_5 Z_2^k, \end{aligned}$$

where $k=1,2,\ldots ,K,$ and $a_3$, $a_4$ and $a_5$ are constants given in Table 4.

**Table 4:** Parameter values in the two-state model for pom1p gradient

Notations	Description	Values
*L*	Length of the Domain	$14\,\upmu \hbox {m}$
$I_1$	Left boundary of $\varOmega _s$	$3.5\, \upmu \hbox {m}$
$I_2$	Right boundary of $\varOmega _s$	$10.5\, \upmu \hbox {m}$
$D_1$	Diffusion coefficient of $S_1$	$0.02\,\upmu \hbox {m}^2\,\hbox {s}^{-1}$
$D_2$	Diffusion coefficient of $S_2$	$0.2\,\upmu \hbox {m}^2\,\hbox {s}^{-1}$
$a_1$	Production parameter of $S_1$	$1.029\,\hbox {s}^{-1}$
$a_2$	Production parameter of $S_2$	$0.441\,\hbox {s}^{-1}$
$a_3$	Fragmentation rate of $S_1$	$0.26 \,\hbox {s}^{-1}$
$a_4$	Aggregation rate	$0.049\,\hbox {s}^{-1}$
$a_5$	Disassociation rate	$0.035\,\hbox {s}^{-1}$
$a_6$	Parameter of production	0.1089
$\varDelta x$	Spatial discretization in $\varOmega _s$	$0.035\,\upmu \hbox {m}$
*h*	Compartment size in $\varOmega _m$	$0.35\,\upmu \hbox {m}$
$\varDelta t$	Time discretization for SPDE	$0.0005\,\hbox {s}$

**Fig. 8:**
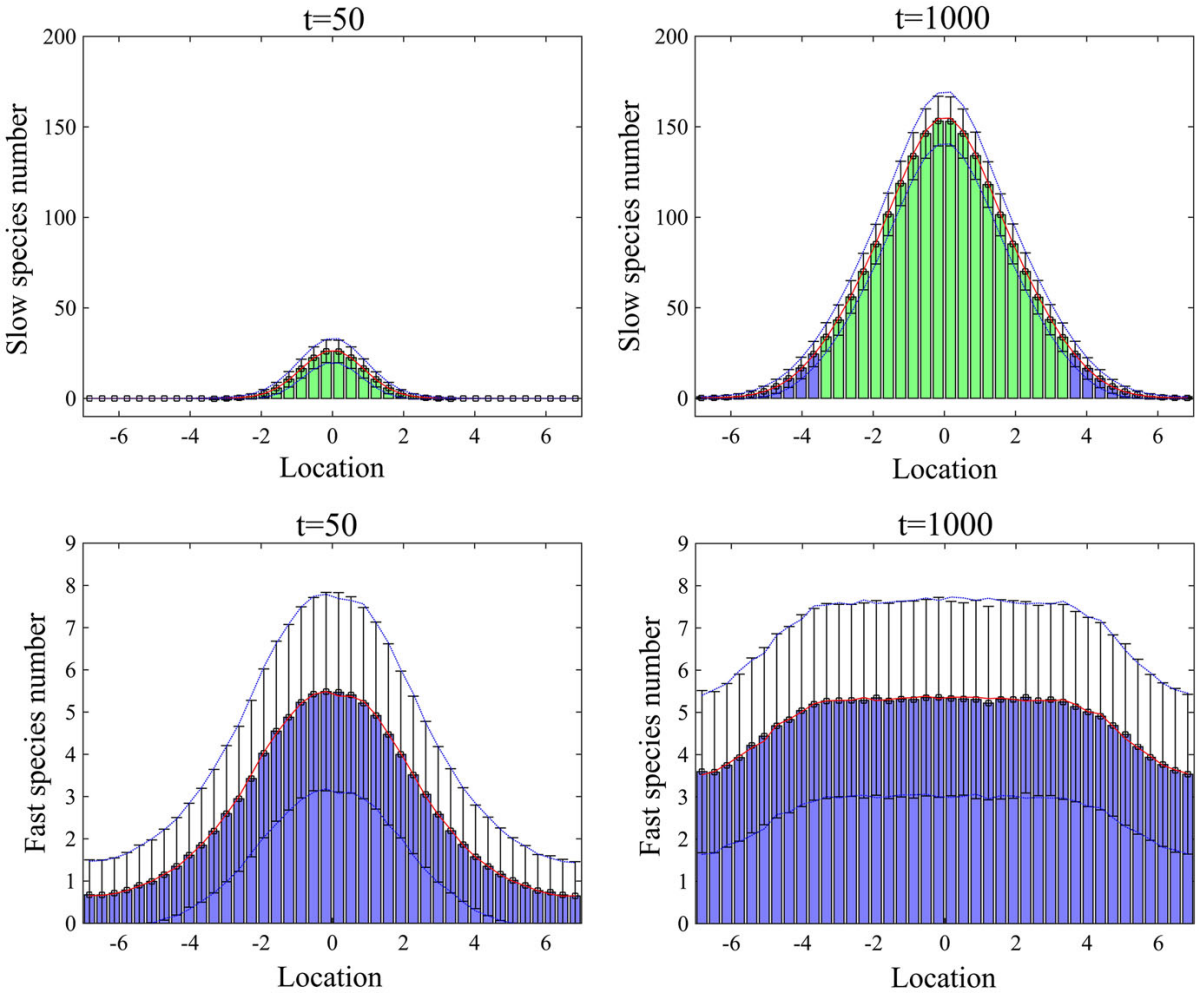
Mean numbers of the molecules of slow-diffusing pom1p clusters, $S_1$ and fast-diffusing pom1p particles, $S_2$ and their standard deviations from the means at $t=50$, $1000\,\mathrm {s}$ computed by averaging over $10^4$ realizations of simulation using the SSA and the multiscale algorithm with Scheme 1 (Color figure online)

In Fig. 8, we present an illustrative simulation of pom1p gradient model. We plot spatial distributions of $S_1$ and $S_2$ at times $t=50\,\hbox {s}$ and $t=1000\,\hbox {s}$. We observe that the spatial distribution of $S_1$ contains a region with high abundance of molecules in the centre of the computational domain. The chemical species $S_2$ has low copy numbers in the entire domain. Therefore, we introduce the SPDE region in the middle of the domain by (note that we fix $K=40$ in this example)

$$\begin{aligned} \varOmega _s=\bigcup _{k=11}^{30} C_k, \end{aligned}$$

where the coarse graining is only applicable to $S_1$ in $\varOmega _s$. In particular, we have introduced two interfaces, $I_1$ and $I_2$ between $\varOmega _s$ and $\varOmega _m$. Diffusion of chemical species $S_1$ is simulated using the algorithm in Table 1. Similarly, production of $S_1$ is implemented using the SPDE and Markov chain model in $\varOmega _s$ and $\varOmega _m$, respectively, as it is done in Eq. (9). The chemical species $S_2$ is simulated by the Markov chain model in the entire domain, because the average number of molecules of $S_2$ is relatively low. In particular, diffusion, production and degradation of $S_2$ are implemented as in the underlying Markov chain model. The only complications are reactions

14 $$\begin{aligned} S_1 \longrightarrow S_2 \qquad \text{ and }\qquad S_1+S_2 \longrightarrow S_1+S_1, \end{aligned}$$

because they include both species $S_1$ and $S_2$, which are in $\varOmega _s$ described by different modelling approaches. We treat these reactions as time-changed Poisson processes in both subdomains $\varOmega _m$ and $\varOmega _s$. Discretizing each compartment, $C_k$, $k=11,12,\ldots ,30$, into $\alpha$ grid points, the state of $S_1$ variable is described by vector, ${{\mathbf {X}}}(t)=(X^1, X^2,\ldots , X^{20(\alpha +1)})$ where $X^1,X^2,\ldots ,X^{10}$ (resp. $X^{20\alpha +11},X^{20 \alpha +12},\ldots ,X^{20(\alpha +1)}$) are the numbers of molecules of $S_1$ in the left (resp. right) part of $\varOmega _m$. The values of SPDE description in compartment $C_k$, $k=11,12,\ldots ,30$, are given by $X^{10+(k-11)\alpha +\ell }$, $\ell =1,2,\ldots ,\alpha$. The state of $S_2$ variable is described by vector, ${{\mathbf {Y}}}(t)=(Y^1, Y^2,\ldots , Y^{40})$ where $Y^k$ is the number of molecules of $S_2$ in compartment $C_k$, $k=1,2,\ldots ,K$. The propensity of the first reaction in (14) of the multiscale model is given by

15 $$\begin{aligned} \lambda _3^{k}({{\mathbf {X}}}) = \left\{ \begin{array}{ll} a_3 X^{k},\; &{}\quad \text{ for } \;k=1,2,\ldots ,10,\\ a_3 \displaystyle \sum _{\ell =1}^{\alpha } X^{10+(k-11)\alpha +\ell },&{}\quad \text{ in }\; \varOmega _s \; \text{(i.e. } \text{ for } \; \text{ k }=11,12,\ldots ,30 \text{) }, \\ a_3 X^{20 (\alpha -1) +k}, &{}\quad \text{ for } \; k=31,32,\ldots ,40. \end{array} \right. \end{aligned}$$

The propensity of the second reaction in (14) of the multiscale model is given by

16 $$\begin{aligned} \lambda _4^{k}({{\mathbf {X}}},Y^k) = \left\{ \begin{array}{ll} a_4 X^{k} Y^k,\; &{}\quad \text{ for } \;k=1,2,\ldots ,10,\\ a_4 Y^k \displaystyle \sum _{\ell =1}^{\alpha } X^{10+(k-11)\alpha +\ell },&{}\quad \text{ in }\; \varOmega _s \; \text{(i.e. } \text{ for } \; \text{ k }=11,12,\ldots ,30 \text{) }, \\ a_4 X^{20 (\alpha -1)+k} Y^k, \; &{}\quad \text{ for } \; k=31,32,\ldots ,40. \end{array} \right. \end{aligned}$$

We simulate reactions in (14) as time-changed Poisson processes with propensities in Eqs. (15)–(16). If the first of these reactions occurs in $C_k$, $k=11,12,\ldots ,30$, we subtract $1/\alpha$ from each $X^{10+(k-11)\alpha +\ell }$, $\ell =1,2,\ldots ,\alpha$, and we add one to $Y^k$. If the second reaction in (14) occurs in $C_k$, $k=11,12,\ldots ,30$, we add $1/\alpha$ to each $X^{10+(k-11)\alpha +\ell }$, $\ell =1,2,\ldots ,\alpha$, and we subtract one from $Y^k$. Note that the conversion of $S_1$ in $C_k$, $k=11,12,\ldots ,30$, is applied equally to the entire $\alpha$ grid points of $C_k$ rather than to one randomly chosen grid point in $C_k$ as it is done (consistently with Eq. (9)) for diffusion across the interfaces.

In Fig. 8, green bars and blue bars represent the mean numbers of molecules of the pom1p clusters and particles in $\varOmega _s$ and $\varOmega _m$ using the multiscale algorithm with **Scheme 1**. Error bars represent one standard deviation from the mean in the multiscale approach. Red lines and blue dotted lines are the mean numbers and their standard deviations from the means computed by the Gillespie SSA simulating the compartment-based approach in the entire domain. Both statistics using the compartment-based approach and the multiscale algorithm are computed by averaging over $10^4$ realizations of the simulations for each case.

## Discussion

A Markov chain model (compartment-based model) has been widely used to describe the discrete nature of the molecular copy numbers and inherent stochasticity in reaction–diffusion systems (Erban and Chapman 2019), but it can be computationally intensive. A possible approach to increase efficiency of simulations is to approximate a part of the model by some coarse-grained methods. In this paper, we have introduced two multiscale algorithms coupling the SPDEs and the Markov chain model, which provide good approximations to the solutions obtained by the Markov chain model applied in the entire spatial domain. Two coupling methods of the Markov chain model and the SPDEs across the interface have been studied. In this section, we compare the presented approach with methods in the literature.

Several Langevin formulations have been introduced to model fluctuating hydrodynamics for chemically reactive species (Bhattacharjee et al. 2015) and stochastic reaction–diffusion systems (Kalantzis 2009; Ghosh et al. 2015). In particular, the spatial chemical Langevin equation was applied to the Gray–Scott model, and its pattern formation was compared to the ones obtained by the reaction–diffusion master equation and PDEs (Ghosh et al. 2015). The spatial chemical Langevin equation consists of a system of stochastic differential equations, and it corresponds to Eq. (4) in Sect. 2. On the other hand, several approaches using SPDEs (Atzberger 2010; Dogan and Allen 2011; Alexander et al. 2002, 2005; Kim et al. 2017) have been introduced to model stochastic reaction–diffusion systems. In Atzberger (2010), the SPDE was derived for reaction–diffusion systems, and discretization of PDEs and stochastic fields was discussed. Unlike Eq. (5), the stochastic fields in the discretized SPDEs account for fluctuations due to diffusion but not for reaction. In Kim et al. (2017), the SPDE for reaction–diffusion systems was derived which is consistent with Eq. (8). In their formulation, diffusion was implemented by the SPDE while the reaction was simulated using the exact or modified SSA.

In Yates and Flegg (2015), two hybrid algorithms are suggested for coupling a compartment-based model and a PDE model when the size of the PDE discretization is less than or equal to the compartment size. Both algorithms extend the PDE approach to the systems with low copy numbers of molecules in a part of the computational domain. The first algorithm considers the PDE solution as the probability density to find a particle within the region and is applied to both cases of low and high copy numbers of molecules in the PDE region. The second algorithm is a simplified and more efficient version of the first one when the PDE region involves the high copy number of molecules. Like in this paper, both algorithms implement a pseudo-compartment with size *h* in the PDE region where *h* represents the compartment size. The second algorithm in Yates and Flegg (2015) is similar to **Scheme 1** if a discretized version of SPDEs replaces the PDEs. However, the interface between the two modelling regimes is assumed to be fixed in Yates and Flegg (2015). In Harrison and Yates (2016), a hybrid algorithm is introduced coupling a compartment-based model and PDEs where the size of the PDE discretization is much finer than the compartment size. In the model, an overlap region is defined with two interfaces (corresponding to the pseudo-compartment in **Scheme 1**) where both modelling regimes are valid, and both cases with fixed and adaptive interfaces are considered. Unlike our pseudo-compartment in **Scheme 1**, the overlap region can contain multiple compartments if needed. On one interface between the compartment-based model and the overlap region, the population of the PDE solution on the interface is matched to the average of the population in the neighbouring compartments. On the other interface between the PDE region and the overlap region, flux on the interface was matched. The hybrid algorithm in Harrison and Yates (2016) approximates the mean population numbers in the compartment-based model if it was possible to apply it over the entire spatial region. The use of the overlap region allows matching the variance between two models in the compartment-based region when the fixed interface is used. On the other hand, the goal of **Scheme 1** and **Scheme 2** is to approximate the compartment-based model by employing the discretized version of SPDEs in the region with high molecules. Therefore, we can match both the mean and variance of the population numbers computed by our multiscale algorithms to the results obtained by the compartment-based model in the whole spatial domain. This is done for both cases with a fixed or adaptive boundary. Unlike the previous approaches in Yates and Flegg (2015); Harrison and Yates (2016), the presented multiscale algorithms can apply to systems with multiple species as it is shown in Sect. 6 where each species has a different partition of the spatial domain into subdomains where different models are used, depending on the spatial distribution of molecules of each species. In Spill et al. (2015), a hybrid algorithm is presented using a compartment-based model and PDEs, where the size of the compartment and numerical discretization for the PDE model is equal.

In this paper, we have discussed the case when the mesh size of the numerical discretization of the SPDEs is smaller (or equal) than the compartment size in the Markov chain model ($h\ge \varDelta x$). This case is useful when we add inherent stochasticity in the PDE model where a fine spatial resolution of the PDE solution is required to describe the solution of the SPDE. This case was also discussed in other hybrid algorithms coupling the compartment-based model and the macroscopic PDEs (Yates and Flegg 2015; Harrison and Yates 2016). The other case, $h<\varDelta x$, discussed, for example, in the hybrid algorithm coupling a random walk on a lattice and the PDE model (Flekkoy et al. 2001), is helpful when the PDE or SPDE model is used as a coarse-grained approximation of the compartment-based model. Such approximation can be used in the region where spatial concentration gradients are not large, so they do not require a fine resolution in space. Although we have focused on the case $h \ge \varDelta x$, the presented approach can be extended to $h<\varDelta x$ as well. In fact, if $h=\varDelta x$, both **Scheme 1** and **Scheme 2** will be the same. If $h<\varDelta x$, we may be able to consider an overlap region (like a pseudo-compartment) in the compartment-based region to extend **Scheme 1**. The presented SPDE-based approach provides a bridge between the stochastic approach (using the Markov chain compartment-based model) and the deterministic approach (using the macroscopic PDEs) by incorporating a discretized version of SPDEs. The SPDEs can be utilized to build other hybrid models, for example, by coupling them with macroscopic PDEs. Then, some approaches used in the hybrid algorithms coupling the compartment-based model with the PDEs (Yates and Flegg 2015; Harrison and Yates 2016; Spill et al. 2015; Smith and Yates 2018) will naturally apply to the case with the SPDEs.
